# The Potential Role of Nanotechnology in Therapeutic Approaches for Triple Negative Breast Cancer

**DOI:** 10.3390/pharmaceutics5020353

**Published:** 2013-06-18

**Authors:** Rebecca Johnson, Nirupama Sabnis, Walter J. McConathy, Andras G. Lacko

**Affiliations:** 1 Institute for Cancer Research, University of North Texas Health Science Center, Fort Worth, TX 76107, USA; E-Mails: rjohnson@live.unthsc.edu (R.J.); nasabnis@yahoo.com (N.S.); 2 LipoMedics LLC., Fort Worth, TX 76107, USA; E-Mail: Walter@Lipo-medics.com (W.J.M.)

**Keywords:** nanoparticle, lipoprotein, triple negative breast cancer, targeted therapy

## Abstract

Triple Negative Breast Cancer, TNBC, a highly aggressive and metastatic type of breast cancer, is characterized by loss of expression of the *estrogen receptor* (*ER*), *progesterone receptor* (*PR*), and a lack of overexpression of the *human epidermal growth factor receptor 2* (*HER2*). It is a heterogeneous group of tumors with diverse histology, molecular uniqueness and response to treatment. Unfortunately, TNBC patients do not benefit from current anti-HER2 or hormone positive targeted breast cancer treatments; consequently, these patients rely primarily on chemotherapy. However, the 5-year survival rate for woman with metastatic TNBC is less than 30%. As a result of ineffective treatments, TNBC tumors often progress to metastatic lesions in the brain and lung. Brain metastases of invasive breast cancer are associated with 1 and 2 year survival rate of 20% and <2% respectively. Because the only current systemic treatment for TNBC is chemotherapy, alternative targeted therapies are urgently needed to improve the prognosis for TNBC patients. This review is focused on opportunities for developing new approaches for filling the current void in an effective treatment for TNBC patients.

## 1. Profiling and Current Therapeutic Approaches for Triple Negative Breast Cancer (TNBC)

Breast cancer is the second leading cause of cancer death among women in the US [1]. TNBC, a highly aggressive and metastatic type of breast cancer, is characterized by loss of expression of the *estrogen receptor* (*ER*), *progesterone receptor* (*PR*), and a lack of overexpression of the *human epidermal growth factor receptor 2* (*HER2*) [2]. Histologically, 77%–90% of TNBC tumors are grade 3 at initial presentation [3,4,5,6] and most patients are under the age of 50 at the onset [7,8,9,10]. TNBC is considered an interval cancer (appearing between mammograms), characterized by overexpression of the tumor suppressor *p53* or a p53 loss-of-function mutation, as well as mutations in retinoblastoma (pRb) and *p16*, G1/S cell cycle regulators [11,12,13,14,15]. TNBC accounts for 15%–20% of all breast cancers [7,8,16,17] with a particularly high prevalence among African-American women. Accordingly 50% of all diagnosed cases of breast cancer among African-American women, in the under 40-age group, are of the TNBC type [18].

Breast cancer represents a varied group of diseases that can be divided into four groups (Table 1), according to their gene-expression profiles (GEP). These are: *luminal A*, *luminal B*, *human epidermal growth factor receptor 2* (*HER2*) amplification, and basal-like [19,20]. Triple negative breast cancer (TNBC) is a subtype of breast cancer that shares with the basal-like group many of its characteristics and GEP markers, including expression of basal *cytokeratins 5/6*, *14*, and *17*, as well as the *epidermal growth factor receptor* (*EGFR*) and *vimentin* [20]. Though 60%–80% of TNBC tumors are classified as basal-like, TNBC is a heterogeneous group with differences in histology, molecular profiles and response to treatment [20]. TNBC is further divided into six subtypes based on their GEP. They are: *basal-like 1*, *basal-like 2*, *immunomodulatory*, *mesenchymal*, *mesenchymal stem-like* and *luminal androgen receptor* [20] (Table 2).

**Table 1:** Breast cancer classification based on gene-expression profile (GEP) characteristics [2,21].

Classes	*ER*	*PR*	*HER2*	*GRADE*	*PROGNOSIS*
*Luminal A*	Pos	Pos	Neg	Low	Good
*Luminal B*	Pos/Neg	Pos/Neg	Pos/Neg	Intermediate/High	Intermediate
*HER2*	Neg	Neg	Pos	High	Poor
*Basal-like*	Neg	Neg	Neg	High	Poor

Pos, Positive; Neg, Negative.

While TNBC and basal like malignancies have significant overlapping features, several differences have also been described [6,10,20,21,22,23,24,25,26,27]. Bertucci *et al.* found that 23% of the tumors they investigated and classified as basal-like via GEP criteria [24,28] did not completely fulfill the TNBC phenotype [20]. Another group representing 29% of those with the TNBC phenotype, were classified as non-basal-like according to GEP criteria. Despite this apparent conflict in classification, there is agreement that TNBC is characterized by loss of expression of the *ER*, *PR*, and a lack of overexpression of *HER2* [29]. Poorly differentiated ductal carcinomas make up 80%–93% of TNBC tumors [6,8,30]. TNBC rarely has a ductal carcinoma *in situ* (DCIS) component, due to the highly invasive nature of this tumor [6,8,31].

**Table 2:** Triple negative breast cancer (TNBC) subtypes based on gene-expression profiles (GEP) [21].

Subtype	GEP
*Basal-like 1 (BL1)*	expresses cell cycle, DNA repair and proliferating genes
*Basal-like 2 (BL2)*	expresses growth factor signaling genes such as EGFR, MET, Wnt, IGF-1R
*Immunomodulatory (IM)*	expresses genes involved in immune cell processes
*Mesenchymal (M)*	expresses genes involved in cell motility, differentiation and EMT processes
*Mesenchymal stem-like (MSL)*	expresses growth factor signaling genes and low levels of proliferating genes
*Luminal androgen receptor (LAR)*	expresses androgen receptor and downstream genes

The overall poor prognosis of TNBC is partly due to its high rate of recurrence and metastases within 5 years of the initial diagnosis [30,32] as well as lack of targeted therapies [33,34]. TNBC tumors do not benefit from current anti-HER2 or hormone positive breast cancer treatments [35] as TNBC patients rely primarily on chemotherapy consisting of either anthracycline-based agents combined with cyclosphosphamide, followed by docetaxel or a combination of docetaxel, doxorubicin and cyclophosphamide [34]. Despite the hypothesis that TNBC would respond well to chemotherapy due to the lack of *HER2* overexpression, these patients have a poorer overall survival than HER2 positive patients [36]. The 5-year survival rate for women with metastatic TNBC is less than 30% [37]. With 1118 patients enrolled, Liedtke *et al.* reported a higher proportion of complete responses with TNBC patients (22%) than with non-TNBC patients (11%). However, the 3-year progressive free survival (PFS) rates and overall survival (OS) were decreased among TNBC patients [37]. These findings may be attributed to a group of TNBC patients with early onset drug resistance [37]. As a result of ineffective treatments, TNBC tumors often progress to metastatic lesions in the brain and lung [38]. Brain metastases of invasive breast cancer are associated with 1 and 2 year survival rate of 20% and <2% respectively [38]. Effective treatment of significant palliative benefit for patients with brain metastases is limited. The classical approach includes whole brain radiation or stereostatic radio surgery [38,39]; however, these treatments do not increase the overall survival of the patient [39], and in some cases has adverse effects on cognitive function [40]. Because the only current systemic treatment for TNBC is chemotherapy, alternative targeted therapies are urgently needed to improve the prognosis for TNBC patients [20]. This review is focused on opportunities for developing new approaches for filling the current void in an effective treatment for TNBC patients.

## 2. Currents Status of TNBC Therapeutics

Currently, the first-line treatment patterns for TNBC include a combination of surgery, radiation, and neoadjuvant/adjuvant chemotherapy, which can often lead to an improved prognosis for early stage TNBC. Kassam et al have demonstrated that, compared to other types of breast cancers, TNBC patients experience a higher proportion of metastatic recurrence (33.9% *vs.* 20.4%; *p* < 0.0001) [41]. Furthermore, in advanced disease, a median overall survival is only 13.3 months, after initial diagnosis [42]. This limited survival validates the urgent need for new approaches as a high priority, compared with other forms of metastatic breast cancers [43,44].

As demonstrated by Liedtke *et al*. groups of patients with TNBC show marked differences with respect to response and prognosis subsequent to neoadjuvant chemotherapy [37]. While some patients with TNBC may benefit from current chemotherapy regimens, there is a sizable group for whom there are only limited benefits. Consequently, four main issues need to be considered for the development of novel therapeutic approaches for TNBC patients: (1) Identification of patients with resistance to current chemotherapy regimens; (2) Development of novel biomarkers to improve the early diagnosis as well as the classification of patients with regards to their respective responses to therapy; (3) Development of alternative strategies for improved bioavailability and targeting of drugs; (4) Improvement of drug delivery vehicles to safely transport the anti-cancer agents to their tumor targets.

**Figure 1:**
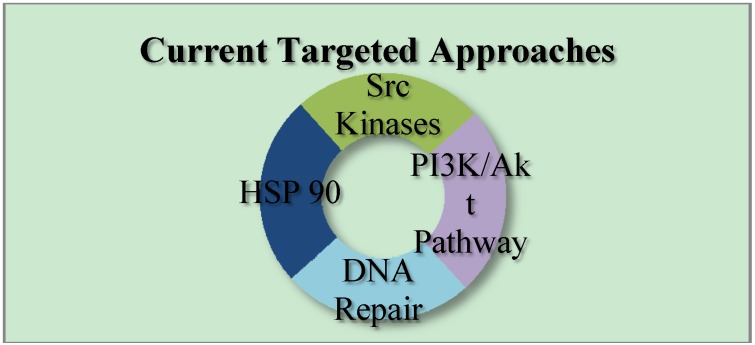
Current therapeutic targets for TNBC treatment.

TNBC patients represent a heterogeneous group with varying molecular profiles and response to treatment [20]. As a result several molecules and signaling pathways are likely targets for new therapeutic approaches. In this review a number of these potential therapeutic targets are highlighted (Figure 1).

## 3. Current Targets for TNBC Therapeutics

### 3.1. PI3K/Akt Pathway

#### 3.1.1. mTOR

**Figure 2:**
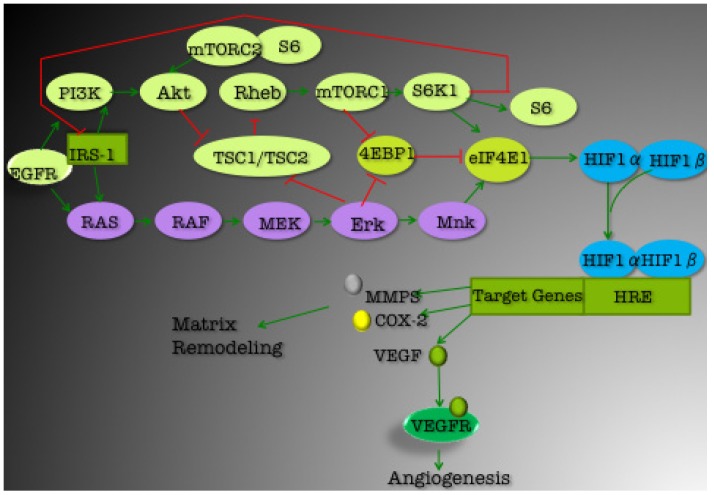
Diagram of PI3K/Akt and Ras/Raf pathway (see text for details).

Eukaryotic translation initiation factor 4E (eIF4E1) along with EGFR have been identified as proteins expressed in brain metastatic cells originating from breast cancer [45]. Once eIF4E1 is activated it also activates hypoxia inducible factor alpha (HIF1α), which then binds with HIF1β, and together they function as transcription factors (TF) for genes involved in angiogenesis, namely matrix metalloproteinases (MMPs) and cyclooxygenase 2 (Cox-2) (Figure 2).

These proteins function together to remodel the extracellular matrix. HIF1α also acts as a TF for the growth hormone VEGF which when bound to its receptor, VEGFR also aids in angiogenesis. The mammalian target of rapamycin (mTOR) is a serine/threonine kinase functioning as a main effector downstream of the phosphatidylinositide 3-kinase (PI3K)/protein kinase B (Akt) pathway [46]. It is involved in many cellular processes including cell growth, survival, and invasion [32,47,48]. mTOR exist in two complexes mTORC1 and mTORC2 [46,47]. mTORC2 phosphorylates Akt at S473 allowing phosphoinositide-dependent kinase 1 (PDK1) to phosphorylate Akt at T308 [47].

Both phosphorylations are needed for Akt activation. Akt is then able to phosphorylate and inhibit tuberous sclerosis 2 (TSC2) keeping it from forming a complex with TSC1 [47]. This drives the GTPase Ras homolog enriched in brain (Rheb) into the GTP bound state. Upon activation, Rheb phosphorylates mTOR at the S2448 position [46,47]. mTORC1 as well as Erk1/2 phosphorylates 4EBP1, the inhibitor of eIF4E1. Phosphorylation of 4EBP1 keeps it from inhibiting eIF4E1 and allows eIF4E1 to promote angiogenesis. mTORC1 also phosphorylates S6K1 which leads to the activation of S6 the small ribosomal subunit. S6 association with mTORC2 allows it to phosphorylate Akt upstream of the mTORC1 complex [46]. Activated S6K1 is a key protein in the negative feedback loop to insulin receptor substrate (IRS-1). When mTOR is inhibited S6K1 does not inhibit IRS-1. IRS-1 can activate both the Ras/Raf and PI3K-Akt pathways as well as activating other receptor tyrosine kinases (RTK), such as VEGFR. In TNBC, KRAS mutations have been reported resulting in constitutive activation of the Ras/Raf pathway [48]. In addition, TNBC patients may also carry gain-of function BRAF mutations further making it harder to effectively target the Ras/Raf pathway [49]. Because rapamycin and its analogues partially inhibit mTORC1 the unobstructed feedback loop eventually overcomes the inhibition and cell proliferation continues again [50]. Rapamycin and its analogs are partial inhibitors of the mTORC1 complex and do not inhibit mTORC2 at all [51]. However; in some cancers the rapamycin analogs have been shown to be very effective at inhibiting cell proliferation at greater than 24 h treatments [47,52,53]. Such results have not been seen with the TNBC cell lines. A recent study on the TNBC cell line MDA-MB-231 has shown that while 72 h of rapamycin treatment induced apoptosis, this effect did not increase above the level of the untreated control [53]. Moreover, combination treatment of rapamycin and the drug indole-3-carbinol actually decreased the level of apoptosis achieved by indole-3-carbinol on its own [53].

A novel ATP-competitive inhibitor of mTOR, Torin1, has been reported to inhibit cell proliferation more effectively than rapamycin [47]. Indeed, studies show that a 10-day treatment of U87 primary glioblastoma multiforme, (GBM) xenografts with Torin 1 resulted in a robust activation of the PI3K/Akt/mTOR pathway and tumor growth suppression by over 99% [54]. Though the signaling mechanism that connects mTOR to autophagy is yet unclear, Torin 1 has been shown to induce autophagy in mouse embryonic fibroblasts (MEF) and HeLa cells [55]. Torin1 has also been shown in decrease protein translation and cause a G1/S cell cycle arrest in MEF cells. Despite its performance Torin 1 is limited in its therapeutic use due to its low bioavailability and half-life of only 0.5 h with *i.v.* administration [54].

#### 3.1.2. EGFR

EGFR is one of the receptor tyrosine kinases (RTK) that is activated by the substrate IRS1 as a result of mTOR inhibition (Figure 2). IRS1 is phosphorylated at S636/639 by the mTOR pathway [55], keeping it from activating RTKs and further activating the PI3K pathway. When mTOR is inhibited this negative feedback loop is disrupted and IRS1 is free to bind with EGFR and other RTKs. EGFRs involvement in cancer growth is well documented [56,57]. Tumors over-expressing EGFR tend to have higher proliferation rates, inhibition of apoptosis, chemoresistance, increased angiogenesis, invasive and metastatic tendencies [58]. Sixty percent of basal-like tumors over-express EGFR and ~70% of TNBC tumors [59,60,61]. These finding make EGFR a reasonable target. 173 patients were treated with cisplatin alone or in combination with cetuximab, an anti-EGFR antibody. The response rate was 20% with those treated with the combination *vs.* 10% with those treated with cisplatin alone [62]. Similar results were seen with the drug combination *vs.* carboplatin alone in a randomized phase II clinical trial of TBCRC001 [28].

#### 3.1.3. IGF1R

The Insulin Growth Factor 1 Receptor (IGF1R) has been associated with the growth, invasion, and metastasis in breast cancer patients and is over-expressed in 50%–75% of TNBCs [63]. IGF1R has been reported to aid in metastasis by allowing the cancer cells to adapt to anchorage-independent growth [64,65]. Indeed pre-clinical trails have shown that over-expressing IGF1R induces tumor formation and metastasis [66,67]. IGF1R has also been shown to inhibit apoptosis induced by chemotherapeutic drugs in the HBL100 breast cancer cell line inferring chemo-resistance to the cancer cells [68].

### 3.2. DNA Repair

#### PARP

BRCA1 (a gene involved in homologous DNA repair) mutations are seen in both basal-like and TNBC type breast cancers. Both subtypes are reported to have a high degree of genetic instability [59,69,70]. 75%–80% of all BRCA1 mutations have been reported to be basal-like by GEP [71,72,73]. BRCA1 mutations have been found in ~60% of the TNBC patients tested [6,74,75,76]. However, BRCA1 silencing due to promoter methylation has also been shown [59]. BRCA1 TNBC patients are among the minority of those who benefit from anthracycline-based chemotherapy, and are also susceptible to platinum based agents [77]. BRCA1 mutations clear the way for alternative DNA repair mechanisms like base excision repair, which relies on Poly(ADP-ribose) polymerase (PARP) [16,59,78]. PARP activation leads to histone acetylation by histone acetyltranferases (HAT) of lysine residues on the *N*-terminus tail of the histone [63], allowing access of the repair machinery to the damaged DNA. PARP inhibition leads to an accumulation of unrepaired DNA damage that would normally be repaired by homologous recombination mediated by BRCA1 [59,79,80]. The resulting abundance of DNA damage induces cell death. Cell death as a result of PARP inhibition and BRCA1 deficiency is known as synthetic lethality [81,82,83,84]. Nonetheless, PARP inhibition is not effective on cancers that lack the BRCA1 mutation.

### 3.3. SRC Kinases

Finn *et al.* evaluated safety and efficacy of dasatinib, an effective SRC-family kinase inhibitor with confirmed preclinical anti-proliferative, anti-metastatic, and anti-osteoclastic activity against TNBC [85]. In a phase II clinical trial of 45 patients with advanced TNBC, as a single agent dasatinib had limited activity; however, the potential benefit of combining dasatinib with various chemotherapeutic drugs is under investigation. In a group of 39 human breast cancer cell lines characterized by gene microarray, basal-type breast cancer cell lines demonstrated the most substantial growth inhibition with dasatinib treatment [86]. Preclinical findings by Tryfonopoulos *et al.* suggest substantial synergy when dasatinib is combined with other agents (specifically, cisplatin and FUDR) in TNBC cell lines [87].

### 3.4. Heat Shock Protein 90

Over-expression of the heat shock protein (HSP) 90 isoforms correlated with a poorer prognosis in certain subtypes of breast cancer including TNBC [88,89], indicating that Hsp90 inhibitors could be used as therapeutic targets against TNBC. This category of agents prevents the protein folding function of the chaperone protein Hsp90, resulting in the degradation of client proteins [90]. A preclinical assessment by Caldas-Lopes *et al.* of the Hsp90 inhibitor PU-H71 in TNBC xenografts indicated substantial antitumor activity [91]. In another study, a combination of Hsp90 inhibitors, tanespimycin and trastuzumab, were shown to be well tolerated and exhibited antitumor activity in patients with breast cancer [91].

### 3.5. Combined Targeted Therapy

As a single agent IGF1R inhibitors have shown limited success against most cancers [63,92,93], combining IGF1R treatment with other targeted therapies may offer an improved therapeutic outcome. Current mTOR inhibition causes the upregulation of the Ras/Raf pathway and inhibition of the negative feedback loop of IRS1 while inhibition of IGF signaling has been shown to inhibit growth and induce death of cancer cells with upregulated PI3K. This effect is, due to a PTEN loss of function mutation, and/or gain of function mutations of the Ras/Raf pathway [94,95]. Combining IGF1R inhibition with mTOR inhibition may thus prove to be effective at inducing cell death in TNBC where the Ras/Raf and/or PI3K pathways are up regulated. Similarly, IGF1R inhibition in combination with EGFR inhibition in EGFR over-expressing cancer cells or IGF1R inhibition in combination with HER2 inhibition in HER2 positive cancer cells have also shown improved results over the use of single agents only [96,97,98]. EGFR inhibition has been shown to sensitize malignant tumors to chemotherapy with cisplatin or carboplatin [99], while combining EGFR inhibition with PARP inhibition has also produced encouraging findings. EGFR inhibition can reduce the expression of the BRCA1 protein, thereby making the cancer cells vulnerable to PARP inhibition [100]. This treatment can allow those TNBC patients without BRCA1 mutations to benefit from PARP inhibition.

As reported on the website clinicaltrials.gov, there are currently 67 clinical trials for TNBC in the U.S. at the time of this review. 53 of these trials are using a combination therapy. Directing combination therapy to the above targets, especially PARP and EGFR, can be effective, as reported by Nowsheen *et al.* [100]. Nevertheless, combination drug therapy can increase the probability of adverse side effects. To circumvent the peripheral toxicity of a combination of chemotherapeutic agents several types of nanoparticles have been developed as drug delivery vehicles [99,100,101].

## 4. Nanoparticles as Drug Delivery Vehicles to Treat TNBC

Early onset of chemoresistance, a hallmark of TNBC tumors [20], contributes to the fact that only 1/3 of TNBC patients have shown a pathological complete response (pCR) after anthracycline or anthracycline + taxane based neoadjuvant chemotherapy [20]. One of the major barriers to successful cancer chemotherapy is the development of multidrug resistance (MDR) within the cell [101,102], often due to the over-expression of the ATP-binding cassette transporter glycoprotein (P-gp) also known as MDR1 [102]. P-gp is an ATP dependent transmembrane drug efflux pump that transports drugs across the cell membrane and out of the cell [102,103]. A number of drugs are substrates for P-gp including the anthracyclines and taxanes that are often used in TNBC treatment [99]. Consequently, drug accumulation in the tumor is limited, underscoring the need for advanced drug delivery vehicles to provide effective alternatives to traditional therapy. Some of these novel drug delivery approaches have been reported to increase the therapeutic index of cytotoxic drugs by prolonging circulating half-life and increasing drug accumulation in the tumor, in addition to reducing the risk of off target effects [103,104].

Patel *et al.*, have developed a non-targeting long circulating liposome to encapsulate tariquidar (XR9576), a P-gp inhibitor that has been used to combat the MDR mechanism with some success [102], along with the microtubule stabilizer paclitaxel. The liposome (with a diameter of 180 to 200 nm) enters the tumor through passive diffusion taking advantage of the increased permeability of the tumor cell environment [102]. Testing their nanoparticle on a taxol resistant ovarian cancer cell line, SKOV-3TR, Patel and colleagues were able to show a decrease in the IC_50_ value of paclitaxel from 2743 nM to 34 nM when treated with the loaded tariquidar liposome *vs.* the free drug [102]. Using pH-sensitive folate targeted micelles loaded with doxorubicin, a common anthracycline; Lee *et al.* have also overcome MDR in the doxorubicin resistant breast cancer cell line MCF-7/DOX^R^ [104]. With an average size of 65 nm these micelles release doxorubicin at acidic pH of 6.8 though some doxorubicin release was observed at pH as high as 7.3 [104].

An As_2_O_3_ precipitate loaded pegylated 100 nm liposome developed by Ahn *et al*. termed Nanobin NB (Ni, As) has had similar effects as the free drug on cell survival, invasion and migration of TNBC cells *in vitro* [105]. The effects exerted by the NB (Ni, As) were mediated partially through caspase activation [105]. Using athymic nude mice as an orthotopic model of TNBC for *in vivo* studies however, showed the vast difference between the free drug and the encapsulated liposome. Using 4 mg/kg of the nanobins *vs.* 4 mg/kg of the free drug given twice weekly by *i.p.* injection, Ahn *et al.*, report that no effect was seen with the free drug while the nanobin significantly inhibited tumor growth and that doubling the free drug concentration to 8 mg/kg did not have an effect at inhibiting tumor growth *in vivo* [105]. The effect put forth by the nanobins was attributed to its ability to infiltrate and remain in the tumor longer than the free drug [105]. This drug depositing liposome has shown some promise for the treatment of TNBC.

A nanobioconjugate designed to deliver anti-EGFR Morpholino antisense oligonucleotides (AON) to the breast cancer tumor was developed by Inoue *et al.* [58]. These nanoparticles have an anti-transferrin receptor monoclonal antibody (mAb) covalently conjugated to a poly (β-L-malic acid) (PLMA) foundation [58], which allows for passage across the membrane. The nanoconjugate is targeted to the cancer cell by the nucleosome specific 2C5 mAb [58]. Using the TNBC cell MDA-MB-468, which over-expresses EGFR, Inoue *et al.* reported that at a 5 µM concentration the nanoconjugate inhibited EGFR expression more significantly than the free AON at 10 µM [58]. *In vivo* studies with athymic nude mice revealed a 56% reduction in tumor volume after 45 days resulting from a robust decrease in EGFR expression and pAkt expression as shown by western blot [58]. Multiple and various biomolecules can be conjugated to the PLMA platform at the same time to reduce tumor size, angiogenesis, invasion and metastasis. Previous reports of these nanoconjugates have stated its ability to inhibit glioma tumor growth and angiogenesis [58], and a variant of the nanoconjugate has also been shown to inhibit tumor growth in HER2 positive breast cancers [58]. This study has also shown the potentially of using this nanoconjugate drug delivery system for the treatment of TNBC.

In recent years RNA interference technology has shown tremendous potential in TNBC therapeutics. A novel siRNA delivery system using polyethyleneimine-coated virus-like particles derived from adeno-associated virus type 2 (PEI-AAV2-VLPs) has been explored for gene delivery recently. Its potential in TNBC treatment is currently under investigation [106]. One of these approaches targeted the enhancer of zeste homolog 2 (EZH2) due to its high expression in breast, prostate, and endometrial cancers [107]. Out of 261 patients with invasive breast cancer 82 were found to have over-expressed EZH2, and the TNBC phenotype [107]. Hussein *et al.* investigated the therapeutic effect of a chitosan nanoparticle loaded with EZH2 siRNA. The MDA-MB-231 cells were injected into the mammary fat pad of female athymic nude mice to produce an orthotopic model of TNBC [107]. After 4 weeks of treatment there was a 73% decrease in tumor weight [107], indicating that EZH2 may be an appropriate therapeutic target for TNBC treatment. In another study a liposomal siRNA preparation, targeted to the eukaryotic elongation factor 2 kinase (eEF-2K) in athymic nude mice, was found to be effective against MDA-MB-231 tumors [108]. In another study nude mice, xenografted with TNBC tumors were treated with the same liposomal preparation resulting in increased sensitization of the tumors to doxorubicin [108].

### Metabolic Profile of TNBC Cells Could Provide New Treatment Opportunities via Biocompatible Nanoparticles

Cancer cells proliferate at a higher rate than normal cells creating a need for malignant cells to acquire sources of energy and cell building constituents far in excess of normal cells. Cancer cells accomplish these tasks via mutating growth factor receptors resulting in constitutive signaling of key metabolic pathways [109]. In addition to basic nutrients, cancer cells have an excessive need for cholesterol for membrane biogenesis [109]. One of the mechanisms that cancer cells use to meet this requirement is by over-expressing the high-density lipoprotein (HDL) SR-B1 receptor [110]. Drug delivery strategies can take advantage of the excessive SR-B1 receptor function in cancer cells and tumors, by utilizing reconstituted high-density lipoproteins (rHDL) that carry anti-cancer agents, instead of cholesterol as their targeted payload [111]. The drug carrying rHDL nanoparticles thus function as a “Trojan Horse” and enhance the therapeutic efficacy of the enclosed drugs toward malignant tumors including TNBC [111]. The over-expression of the SR-B1 in malignant tissues [110] has the potential to facilitate the enhanced selective delivery of anti-cancer agents to tumors [110,111] thus providing a marked improvement of the current chemotherapy regimens, including limiting off target toxicity [110]. Though the above-mentioned nanoparticles have potential, at the time of the review none of them are in clinical trials.

## 5. Conclusion

TNBC is a heterogeneous group of cancers with diverse histology, molecular profile and response to treatment [20]. Characterized by loss of expression of the estrogen receptor (ER), progesterone receptor (PR), and a lack of over-expression of the human epidermal growth factor receptor 2 (HER2), TNBC is a highly aggressive and metastatic disease with a very overall poor prognosis with a current five-year survival rate of less than 30%. Because the current therapeutic modalities for TNBC have only limited effectiveness, alternative therapies are urgently needed to improve the prognosis for TNBC patients. This review focuses on the potential of nanoparticles as effective enhancers of treatment for TNBC tumors. Some of the current nano-delivery formulations should have the ability to vastly improve the response rate of TNBC patients by transporting the anti-cancer agents selectively to the tumors while bypassing MDR mechanisms. In addition, some of these nanoparticles have the capacity to reduce the exposure of potentially harmful drugs to the non-malignant surrounding tissues and thus markedly reduce the off target effects of chemotherapy.
